# Methotrexat-Versorgung vor dem Einsatz von Biologika bei rheumatoider Arthritis: Eine Analyse der Leitliniengerechtigkeit

**DOI:** 10.1007/s00393-021-01086-0

**Published:** 2021-09-20

**Authors:** Nicolas Pardey, Jan Zeidler, Tim Fritz Nellenschulte, Jona T. Stahmeyer, Kirsten Hoeper, Torsten Witte

**Affiliations:** 1 Center for Health Economics Research Hannover (CHERH), Leibniz Universität Hannover, Otto-Brenner-Str. 7, 30159 Hannover, Deutschland; 2 Arzneimittelversorgung, Mobil Krankenkasse, Hannover, Deutschland; 3 Stabsbereich Versorgungsforschung, AOK Niedersachsen, Hannover, Deutschland; 4 Klinik für Rheumatologie und Immunologie, Medizinische Hochschule Hannover, Hannover, Deutschland

**Keywords:** Unterversorgung, bDMARD, Verordnung, Ausschöpfung, GKV-Routinedaten, Underuse, bDMARD, Prescription, Exhaustion, Health insurance claims data

## Abstract

**Hintergrund:**

Seit vielen Jahren erweitern Biologika die Therapieoptionen bei rheumatoider Arthritis (RA). Der Einsatz dieser biologischen „disease-modifying antirheumatic drugs“ (bDMARDs) ist gemäß deutscher und europäischer Behandlungsleitlinien jedoch erst bei Ausschöpfung der Methotrexat (MTX)-Erstlinientherapie von mindestens 20 mg/Woche indiziert. Ziel der Studie ist es, die Leitliniengerechtigkeit der MTX-Verordnung im ambulanten Sektor vor der Biologikatherapie zu überprüfen.

**Methodik:**

Es wurden Routinedaten der AOK-Niedersachsen der Jahre 2013 bis 2016 für alle Versicherten zur Verfügung gestellt, die im Studienzeitraum eine RA-Diagnose sowie eine bDMARD-Verordnung aufweisen. Innerhalb eines patientenindividuellen Beobachtungszeitraums von 180 Tagen vor der ersten bDMARD-Verordnung wurde die maximal verordnete MTX-Dosierung untersucht.

**Ergebnisse:**

Die Studienpopulation umfasst 405 Patienten (90 inzident, 315 prävalent). Bei 60,0 % der inzidenten Patienten und 67,0 % der prävalenten Patienten wurde eine maximale MTX-Verordnung von < 20 mg/Woche beobachtet. Männer weisen im Mittel mit 17,1 ± 4,8 mg eine höhere MTX-Maximaldosierung als Frauen auf (14,9 ± 5,0 mg; *p* < 0,0001); 29,6 % der Studienpopulation erhielten im Beobachtungszeitraum ausschließlich orale Verordnungen. Umstellungen auf eine parenterale Applikationsform wurden bei 12,4 % der Patienten festgestellt.

**Diskussion:**

Ein gezielter Einsatz des gesamten vorgesehenen Therapiespektrums vor der Initiierung einer bDMARD-Therapie kann zu einer kosteneffizienten Versorgung der RA beitragen. Die Studie zeigt Indizien für mögliche Defizite in der ambulanten MTX-Verordnungspraxis auf und kann für eine effiziente Therapie sensibilisieren.

**Zusatzmaterial online:**

Die Online-Version dieses Beitrags (10.1007/s00393-021-01086-0) enthält die Tabellen S1–S3.

## Abstract (en)

**Background:**

With the introduction of biologics the treatment landscape for patients with rheumatoid arthritis (RA) has rapidly expanded; however, according to German and European treatment guidelines the use of biologic disease-modifying antirheumatic drugs (bDMARD) is only indicated after insufficient response under methotrexate (MTX) doses of at least 20 mg/week (first-line treatment). The aim of the study was to analyze the guideline compliance of MTX prescription in the outpatient sector prior to treatment with biologics.

**Material and methods:**

Claims data from the AOK Lower Saxony from 2013 to 2016 were provided for all insured patients with a diagnosis of RA and bDMARD prescription during the study period. Within a patient-specific observational period of 180 days prior to the first bDMARD prescription, the maximum prescribed MTX dosage was examined.

**Results:**

Data from 90 incident and 315 prevalent RA patients were analyzed. A maximum MTX prescription of < 20 mg/week was observed in 60.0% of incident patients and in 67.0% of prevalent patients. Men had a higher mean MTX maximum dose (17.1 ± 4.8 mg) than women (14.9 ± 5.0 mg; *p* < 0.0001). Of the study population 29.6% received oral only prescriptions during the observational period. In 12.4% of patients a switch to parenteral administration was made.

**Discussion:**

Targeted use of the full spectrum of therapies provided prior to initiation of bDMARD treatment may contribute to cost-effective RA care. This study showed indications for potential deficits in outpatient MTX prescription practice and can raise awareness for efficient treatment.

Bei der Behandlung der rheumatoiden Arthritis (RA) kommen neben der Standardbasistherapie mit Methotrexat (MTX) bei anhaltend hoher Krankheitsaktivität in den letzten Jahren vermehrt auch Biologika zum Einsatz. Internationale Studien haben gezeigt, dass Methotrexat vor der Einleitung einer Biologikatherapie teilweise in zu geringen Dosierungen verabreicht wird. In dieser Studie wird untersucht, ob das in den Leitlinien für die Basistherapie mit Methotrexat definierte Therapiespektrum im deutschen Versorgungskontext voll ausgeschöpft wird.

## Hintergrund und Fragestellung

Die rheumatoide Arthritis (RA) stellt mit einer Prävalenz von ca. 0,8 % der erwachsenen Bevölkerung in Deutschland die häufigste chronisch inflammatorische Erkrankung dar [28]. Seit einigen Jahren gilt Methotrexat (MTX) aufgrund seiner langfristigen Wirksamkeit und des günstigen Risikoprofils bei der Therapie der RA als „disease-modifying antirheumatic drug“ (DMARD) erster Wahl [17, 21]. Dabei wird MTX sowohl in Monotherapie, als auch in Kombinationstherapie mit anderen klassischen synthetischen DMARDs (csDMARDs), biologischen DMARDs (bDMARDs) oder „targeted“-synthetischen DMARDs (tsDMARDs) eingesetzt und insbesondere in der Phase nach der Diagnosestellung der RA in Kombination mit Glukokortikoiden [22]. Die optimale Erstdosierung des MTX wird auf bis zu 15 mg/Woche in oraler Form beziffert, eine rasche Eskalation auf bis zu 25 und teilweise sogar bis 30 mg/Woche sowie die Umstellung auf eine parenterale Applikation werden bei Verträglichkeit und dem Ausbleiben einer Remission angeraten [25]. Auch die aktuellen Leitlinien der European Alliance of Associations for Rheumatology (EULAR) sowie die deutsche S2e-Leitlinie (2018) empfehlen eine Dosiseskalation auf bis zu 25 mg/Woche. Weiterhin sollte MTX ab einer Dosierung von > 15 mg/Woche aufgrund der höheren Bioverfügbarkeit in parenteraler Form appliziert werden. Auf die Option einer Dreifachkombination aus MTX, Sulfasalazin und Hydroxychloroquin bei fehlenden negativen Prognosefaktoren wird in den deutschen Leitlinien zur medikamentösen Behandlung der RA (S1- und S2e-Leitline) hingewiesen [6, 9]. Erst bei Vorliegen ungünstiger Prognosefaktoren wird übereinstimmend die Hinzunahme eines bDMARDs oder tsDMARDs zur MTX-Therapie empfohlen [6, 9, 22]. Studien aus den USA legen jedoch nahe, dass bDMARDs häufig bereits bei MTX-naiven Patienten eingesetzt werden oder MTX in nur geringen Dosen verabreicht wird, bevor eine Therapieumstellung auf ein bDMARD erfolgt [14, 18].

Ziel dieser Studie ist es, für den deutschen Versorgungskontext zu untersuchen, ob RA-Patienten im Hinblick auf verordnete MTX-Dosierungen und -Applikationsformen leitliniengerecht behandelt werden, bevor eine Umstellung auf eine bDMARD-Mono- oder Kombinationstherapie erfolgt. Die Grundlage bildet die im Studienzeitraum gültige deutsche S1-Leitlinie zur medikamentösen Therapie der RA. Diese empfiehlt spätestens 6 Monate nach Behandlungsbeginn eine Eskalation der MTX-Dosierung auf mindestens 20 mg/Woche [9]. Weiterhin soll untersucht werden, welcher Anteil der bDMARD-Patienten eine Umstellung der MTX-Applikationsform zugunsten einer parenteralen Abgabe aufweist. Nach unserer Kenntnis liegen für den deutschen Versorgungskontext bisher keine Studienergebnisse über die maximal verordneten MTX-Dosierungen im Vorfeld der Umstellung auf eine bDMARD-Therapie vor. Die Ergebnisse können Defizite in der ambulanten Verordnungspraxis aufzeigen und zur Sensibilisierung einer leitliniengerechten MTX-Therapie, auch im Hinblick auf die Kosteneffektivität der Versorgung im ambulant ärztlichen Bereich, beitragen.

## Studiendesign und Untersuchungsmethoden

### Datenbasis

Für die Studie standen Routinedaten der AOK Niedersachsen (AOKN) für den Studienzeitraum vom 01.01.2013 bis 31.12.2016 zur Verfügung. Die Kriterien zum Einschluss in die Studienpopulation sind erstens die Dokumentation mindestens einer gesicherten ambulanten oder stationären Diagnose der RA und zweitens die Verordnung mindestens eines bDMARDs im Studienzeitraum (Tabelle S1 im Online-Zusatzmaterial). Die RA-Diagnosen schließen dabei die ICD-10-GM Kodierungen M05: „Seropositive chronische Polyarthritis“ und M06: „Sonstige chronische Polyarthritis“ ein. Neben den Stammdaten, welche Informationen zu Alter und Geschlecht der Versicherten beinhalten, wurden alle Haupt- und Nebendiagnosen des stationären Sektors sowie alle ambulanten Diagnosen der Studienpopulation für den Studienzeitraum übermittelt. Daten zur ambulanten Arzneimittelverordnung wurden in Form der Anatomisch-Therapeutisch-Chemischen (ATC) Klassifikation und der Pharmazentralnummer (PZN) zur Verfügung gestellt, sofern es sich um Arzneimittel zur Behandlung der RA handelte.

### Studiendesign

Die erste ambulant oder stationär dokumentierte Diagnose der RA im Studienzeitraum, im Folgenden als Diagnoseindex bezeichnet, stellt das individuelle Einschlussdatum des Versicherten in die Studie dar. Da ambulante Diagnosen in Routinedaten nur quartalsweise abgebildet werden, wurde der Diagnoseindex auf den 1. Tag des Diagnosequartals festgelegt. Weiterhin wurde ein bDMARD-Index definiert, der datumsgenau die erstmalige Verordnung eines bDMARDs angibt. Für jeden Patienten wurde ein individueller Beobachtungszeitraum definiert, welcher rückwirkend ab bDMARD-Index einen Zeitraum von 180 Tagen abdeckt. Innerhalb dieses Zeitraums von 6 Monaten soll die medikamentöse Therapie gemäß der S1-Leitlinie bei unzureichendem Ansprechen auf MTX-Mono- und Kombinationstherapien auf ein bDMARD umgestellt werden. Die Behandlungsdauer wird als Differenz des bDMARD-Index und des Diagnoseindex definiert. Um für alle Patienten einen 6‑monatigen Beobachtungszeitraum zu gewährleisten, wurde das Jahr 2013 als Wash-out-Periode gewählt. Somit können Patienten identifiziert und von der Studienpopulation ausgeschlossen werden, deren bDMARD-Index im Jahr 2013 liegt. Diese Periode ermöglicht ebenfalls die Differenzierung von inzidenten und prävalenten Patienten, wobei Patienten, deren erste beobachtbare RA-Diagnose bereits im Jahr 2013 vorliegt, als prävalent definiert werden. Zur Nachbeobachtung der MTX-Medikation nach dem bDMARD-Index wurde ebenfalls ein Zeitraum von 180 Tagen herangezogen.

Die Patienten wurden, in Abhängigkeit der im individuellen Beobachtungszeitraum kodierten Diagnosen in die Diagnosegruppen M05.-, M06.- oder M05.-/M06.- bei Vorliegen beider Diagnosen eingeteilt. Das Alter der Patienten wurde zum Zeitpunkt der ersten beobachtbaren RA-Diagnose erhoben. In einer Subgruppenanalyse wurden niereninsuffiziente Patienten (ICD-10-GM N18.2–N18.5, N18.9) identifiziert, da aufgrund der Kontraindikation von einer geringeren MTX-Maximaldosis ausgegangen wurde. Der Einschluss erforderte mindestens eine entsprechende Diagnose im Zeitraum von bis zu 1 Jahr vor Beginn des individuellen Beobachtungszeitraums.

Die verordneten MTX-Dosierungen und -Applikationsformen wurden mithilfe der PZN unter Verwendung der Lauer-Taxe Online 4.0 ermittelt. In der vorliegenden Studie wird die Applikationsform nach oral (Tablette) und parenteral (Spritze, Injektionslösung, Fertigpen) differenziert. Die Dosierungen wurden in die Gruppen 2,5–7,5 mg, 10–12,5 mg, 15–17,5 mg, 20–22,5 mg und ≥ 25 mg eingeteilt. Anschließend wurde für jeden Patienten die maximal verordnete Dosierung im individuellen Beobachtungszeitraum ermittelt. Patienten, die in diesem Zeitraum mindestens 1‑mal von einer oralen zu einer parenteralen Applikationsform oder von einer parenteralen zu einer oralen Applikationsform gewechselt haben, wurden als Switcher definiert. Durchgeführt wurde die Datenanalyse mit SAS 9.4 für Windows, SAS Institute Inc., Cary, NC, USA. Die berichteten *p*-Werte beziehen sich auf den t‑Test für unabhängige Stichproben und die einfaktorielle Varianzanalyse. Varianzhomogenität wurde mit dem Levene-Test überprüft. Als statistisch signifikant wird ein *p*-Wert von ≤ 0,05 angenommen.

### Ausschlusskriterien

**Figure 1:**
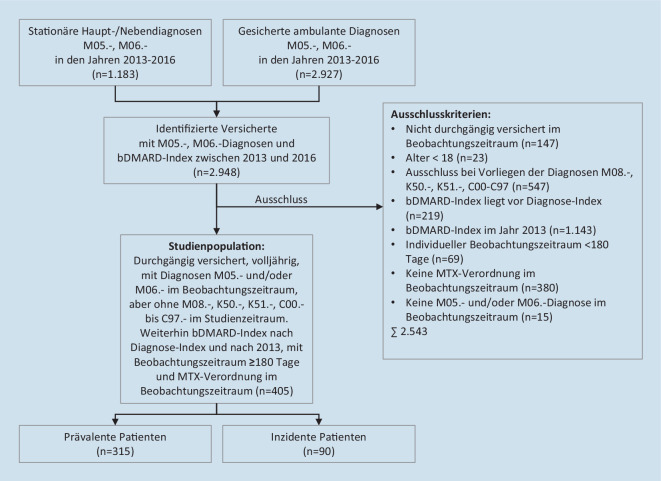

Der Aufgriff der Studienpopulation ist der Abb. 1 zu entnehmen. Von der Studie ausgeschlossen wurden Versicherte, die in den Jahren 2013 bis 2016 nicht durchgängig (≥ 330 Tage pro Jahr) bei der AOKN versichert waren oder zu Beginn des Studienzeitraums nicht mindestens 18 Jahre alt waren. Weiterhin wurden Versicherte ausgeschlossen, sofern mindestens eine ambulante oder stationäre Diagnose der ICD-10-GM in Form einer juvenilen Arthritis (M08.-), Morbus Crohn (K50.-), Colitis ulcerosa (K51.-) oder einer malignen Tumorerkrankung (C00.- bis C97.-) im Studienzeitraum kodiert wurde. Da diese Erkrankungen medikamentös regelmäßig auch mittels Biologika therapiert werden, soll ausgeschlossen werden, dass die Verordnung von Biologika nicht ursächlich auf die Behandlung der Studienindikationen abzielte. Ebenfalls ausgeschlossen wurden Versicherte, deren bDMARD-Index zeitlich entweder vor dem studienspezifischen Diagnoseindex liegt oder deren bDMARD-Index in der Wash-out-Periode liegt, da für diese Patienten keine ausreichende Beobachtung der MTX-Medikation vor dem bDMARD-Index besteht. Versicherte, deren individueller Beobachtungszeitraum nicht mindestens 180 Tage betrug oder denen in diesem Zeitraum nicht mindestens 1‑mal MTX zulasten der GKV verordnet wurde oder keine RA-Diagnose kodiert wurde, wurden ebenfalls ausgeschlossen.

## Ergebnisse

Die Charakteristika der Studienpopulation sind in Tab. 1 dargestellt. Die Studienpopulation umfasst 405 Patienten, wobei Frauen mit einem Anteil von 64,9 % die Mehrheit darstellen. Das Durchschnittsalter beträgt 52,2 ± 11,9 Jahre. Mit 196 Patienten stellt die M05.-/M06.-Gruppe die größte Gruppe, die Diagnosegruppe M05.- (*n* = 47) die kleinste Gruppe dar. Als inzidente Patienten wurden 90 Versicherte definiert. Das Durchschnittsalter (49,7 ± 13,6 Jahre) ist etwas geringer als in der prävalenten Studienpopulation. Die mediane Behandlungsdauer der inzidenten Patientengruppe beträgt 409 Tage.

**Table 1:** 

	Inzident (*n* = 90)	Prävalent (*n* = 315)	Gesamt (*n* = 405)
**Geschlecht**			
Männlich *n* (%)	35 (38,9 %)	107 (34,0 %)	142 (35,1 %)
Weiblich *n* (%)	55 (61,1 %)	208 (66,0 %)	263 (64,9 %)
**Alter**			
MW (SD)	49,7 (±13,6)	52,9 (±11,4)	52,2 (±11,9)
Median (Min, Max)	51 (20, 83)	52 (22, 81)	52 (20, 83)
**Behandlungsdauer (in Tagen)**^a^			
*Alle Diagnosegruppen*	*n* *=* *90*	*n* *=* *315*	*n* *=* *405*
MW (SD)	455,9 (±201,3)	864,5 (±328,1)	773,7 (±348,6)
Median (Min, Max)	409 (190, 1030)	867 (185, 1456)	716 (185, 1456)
*M05*	*n* *=* *13*	*n* *=* *34*	*n* *=* *47*
MW (SD)	500,5 (±258,1)	901,8 (±328,8)	790,8 (±357,6)
Median (Min, Max)	370 (243, 1030)	923,5 (315, 1431)	764 (243, 1431)
*M06*	*n* *=* *36*	*n* *=* *126*	*n* *=* *162*
MW (SD)	411,2 (±167,3)	833,2 (±325,8)	739,4 (±345,6)
Median (Min, Max)	386,5 (200, 807)	853 (185, 1456)	668,5 (185, 1456)
*M05/M06*	*n* *=* *41*	*n* *=* *155*	*n* *=* *196*
MW (SD)	481,1 (±206,5)	881,8 (±329,8)	798,0 (±348,4)
Median (Min, Max)	481 (190, 873)	869 (203, 1442)	753,5 (190, 1442)

*Max* Maximum, *Min* Minimum, *MW* Mittelwert, *SD* „standard deviation“ (Standardabweichung)

^a^Die Behandlungsdauer der prävalenten Studienpopulation hat aufgrund der Unbeobachtbarkeit des Diagnoseindex eine eingeschränkte Aussagekraft

Die höchste verordnete MTX-Dosierung im Beobachtungszeitraum beträgt für 6,2 % der gesamten Studienpopulation 2,5–7,5 mg. Eine maximale Dosierung von 10–12,5 mg wurde für 25,4 % der Patienten beobachtet, 33,8 % der Studienpopulation wurde eine Maximaldosierung von 15–17,5 mg verordnet, während 26,2 % eine Dosierung von 20–22,5 mg erhielten. Eine Maximaldosierung von mindestens 25 mg erreichten 8,4 % der Studienpopulation (Tab. 2). In der Gruppe der inzidenten Patienten erhielten 54 Personen (60,0 %) eine Maximaldosierung von unter 20 mg. In der Gruppe der prävalenten Patienten (*n* = 315) liegen 211 Patienten (67,0 %) unter diesem Grenzwert. Verglichen mit prävalenten Patienten (15,4 ± 5,0 mg), wurde inzidenten Patienten (16,5 ± 5,3 mg) im Mittel keine signifikant höhere Maximaldosierung verordnet (*p* = 0,0705)^1^. Männer weisen durchschnittlich mit 17,1 ± 4,8 mg eine signifikant höhere MTX-Dosierung als Frauen auf (14,9 ± 5,0 mg, *p* < 0,0001). In Abhängigkeit der Diagnosegruppe (*p* = 0,4793) oder der Altersgruppe (*p* = 0,1253) zeigen sich hingegen keine signifikanten Abweichungen. Bei 284 Patienten (70,1 %) wurde die maximale MTX-Dosierung in parenteraler Form verordnet. Dosierungen von über 15 mg wurden ausschließlich parenteral appliziert, wohingegen Maximaldosierungen bis 12,5 mg überwiegend in oraler Applikationsform verordnet wurden.

**Table 2:** 

	Inzident (*n* = 90)			Prävalent (*n* = 315)			Gesamt (*n* = 405)
	Oral	Parenteral	Gesamt	Oral	Parenteral	Gesamt	
MTX-Dosierung	*n* (%)	*n* (%)	*n* (%)	*n* (%)	*n* (%)	*n* (%)	*n* (%)
2,5–7,5 mg	3 (3,3)	2 (2,2)	5 (5,6)	12 (3,8)	8 (2,5)	20 (6,3)	25 (6,2)
10–12,5 mg	14 (15,6)	4 (4,4)	18 (20,0)	69 (21,9)	16 (5,1)	85 (27,0)	103 (25,4)
15–17,5mg^a^	3 (3,3)	28 (31,1)	31 (34,4)	20 (6,3)	86 (27,3)	106 (33,7)	137 (33,8)
20–22,5 mg	–	25 (27,8)	25 (27,8)	–	81 (25,7)	81 (25,7)	106 (26,2)
≥ 25mg^b^	–	11 (12,2)	11 (12,2)	–	23 (7,3)	23 (7,3)	34 (8,4)

*MTX* Methotrexat

^a^Oral verordnete Dosierungen in dieser Gruppe betragen ausschließlich 15 mg

^b^27,5 mg wurden nicht verordnet. Eine inzidente Person erhielt 30 mg

Gemäß Studiendesign haben alle Patienten im Beobachtungszeitraum mindestens eine MTX-Verordnung erhalten. Darüber hinaus weisen 205 Patienten Verordnungen von Leflunomid und/oder Sulfasalazin auf. Da eine Kombinationstherapie aus MTX + Leflunomid bzw. Sulfasalazin möglicherweise eine geringere MTX-Maximaldosierung zur Folge haben könnte, wurde eine Sensitivitätsanalyse durchgeführt. Es zeigte sich, dass diese Subgruppe im Mittel keine signifikant geringere Maximaldosierung aufweist (*p* = 0,6699) und der Anteil der Patienten mit einer Maximaldosis von < 20 mg/Woche mit 62,4 % nahezu identisch ist (Tabelle S3 im Online-Zusatzmaterial).

Weiterhin wurden in der Studienpopulation 22 niereninsuffiziente Patienten identifiziert. Diese Subgruppe weist im Vergleich zur gesamten Studienpopulation ein höheres Durchschnittsalter von 65,3 ± 10,0 Jahren auf (*p* < 0,0001). Mit 14,4 ± 5,5 mg/Woche ist die durchschnittliche Maximaldosierung jedoch nicht signifikant geringer (*p* = 0,2340).

Die im Beobachtungszeitraum letzte MTX-Verordnung vor (oder in Kombination mit) einem bDMARD wurde bei 284 Patienten parenteral, bei 121 Patienten oral appliziert. Dabei erhielten 257 Patienten (63,5 %) der Studienpopulation die MTX-Verordnungen über den gesamten Beobachtungszeitraum in ausschließlich parenteraler Form, während das MTX bei 120 Patienten (29,6 %) ausschließlich oral appliziert wurde. Switches von einer oralen zu einer parenteralen Applikationsform wurden bei 17 Patienten (4,2 %), von einer parenteralen zu einer oralen Applikationsform, bei 13 Patienten (3,2 %) festgestellt (Tab. 3). Damit ergibt sich ein Switching-Anteil von oraler zu parenteraler Applikation von 12,4 %, bezogen auf die nicht ausschließlich parenteral behandelten Patienten. Die ausschließlich parenterale Applikation führte dabei im Mittel zu einer Maximaldosis von 17,7 ± 4,4 mg, die ausschließlich orale Applikation war mit 10,6 ± 2,3 mg hingegen signifikant geringer. Die Dosierungssteigerung nach dem Switch auf eine parenterale Applikation beträgt durchschnittlich 9,3 ± 4,8 mg (Ergebnisse nicht dargestellt).

**Table 3:** 

	Inzident (*n* = 90)		Prävalent (*n* = 315)		Gesamt (*n* = 405)	
MTX-Applikationsform	*n*	%	*n*	%	*n*	%
Ausschließlich oral	19	21,1	101	32,1	120	29,6
Ausschließlich parenteral	63	70,0	194	61,6	257	63,5
Oral zu parenteral^a^	4	4,4	13	4,1	17	4,2
Parenteral zu oral^a^	6	6,7	7	2,2	13	3,2

*MTX* Methotrexat

^a^Mehrfachswitches sind möglich

## Diskussion

In dieser Studie wurde die MTX-Verordnungspraxis im ambulanten Sektor auf der Grundlage einer Routinedatenanalyse untersucht. Eine leitliniengerechte Therapie der RA verlangt eine MTX-Eskalation auf mindestens 20 mg/Woche, bevor wegen nicht ausreichender Wirksamkeit der Einsatz eines Biologikums empfohlen wird. In Übereinstimmung mit US-Studien konnte gezeigt werden, dass MTX-Dosierungen vor einer bDMARD-Anschlusstherapie bei einem großen Teil der Patienten auch im deutschen Versorgungskontext nicht ausgeschöpft wurden [14, 18]. Bei 60,0 % der inzidenten Patienten wurde eine Maximaldosierung von unter 20 mg/Woche beobachtet, während 67,0 % der prävalenten Patienten diesen Grenzwert unterschreiten. Eine MTX-Medikation wurde im Rahmen der bDMARD-Anschlusstherapie bei 72,6 % der Patienten beibehalten.

Patientenindividuelle Gründe für den Einsatz eines bDMARD bei unzureichender MTX-Dosierung lassen sich mithilfe von Routinedaten nicht näher untersuchen, da keine klinischen Parameter, wie z. B. Laborwerte, erfasst werden [13]. Besonders gastrointestinale Unverträglichkeiten, Hepatotoxizität und Störungen der Blutbildung erfordern bei einem Teil der Patienten eine Therapieanpassung im Hinblick auf die MTX-Dosierung und -Applikationsform oder die Umstellung auf alternative cs-Therapien oder den Einsatz von bDMARD-Therapien [10, 20]. In diesem Zusammenhang ist ebenfalls zu berücksichtigen, dass eine unzureichende Supplementierung von Folsäure die MTX-Verträglichkeit zusätzlich negativ beeinflusst und somit zu einem früheren Einsatz eines bDMARD führen kann [20]. Dabei genügt die Supplementierung von 10 mg/Woche Folsäure auch bei gesteigerten MTX-Dosierungen bei der Behandlung der RA [4]. In der vorliegenden Studie wurden keine Daten zur Verordnung von Folsäuresupplementen erhoben. Eine Routinedatenanalyse für den deutschen Versorgungskontext aus dem Jahr 2012 zeigt jedoch, dass lediglich ca. 65 % der mit MTX therapierten Patienten auch Folsäure verordnet wurde [27].

In der vorliegenden Studie zeigte sich mit 29,6 % der Studienpopulation ein hoher Anteil von Patienten, denen MTX ausschließlich in oraler Applikationsform vor Einsatz eines bDMARD verordnet wurde. Der Switching-Anteil von oraler zu parenteraler Applikation, bezogen auf die nicht ausschließlich parenteral behandelten Patienten, ist mit 12,4 % ebenfalls gering. Im Vergleich zu oralem MTX weist die parenterale Applikation bei gleicher Dosierung, besonders bei > 15 mg/Woche, eine höhere Bioverfügbarkeit auf und steigert trotz höherer Dosierungen evtl. die Verträglichkeit. Die Notwendigkeit der Gabe eines bDMARD ist bei parenteraler Applikation somit in geringerer Häufigkeit gegeben [11, 19]. Weiterhin zeigt parenterales MTX sowohl bei initialer Verordnung als auch bei Switch von einer oralen Applikation eine höhere Wirksamkeit in Bezug auf DAS28-, ACR20- und ACR70-Scores und könnte folglich zu einer Ausreizung der MTX-Therapie beitragen [2, 8]. Bei den ausschließlich oral behandelten Patienten bestünde die Switching-Option auf eine parenterale Applikation. Bei diesem Switch wurde eine Steigerung der durchschnittlichen Dosierung von 9,3 ± 4,8 mg festgestellt. Das Switching-Verhalten wurde jedoch nur im Beobachtungszeitraum untersucht, vorherige Switches werden nicht abgebildet.

Bei Verfehlen der Treat-to-target (T2T)-Ziele einer MTX-Ersttherapie bestehen die Therapieoptionen einer Dreifachkombination (MTX/Sulfasalazin/Hydroxychloroquin) oder bei ungünstigen Prognosefaktoren eine Kombinationstherapie aus MTX und einem bDMARD [9]. Beide Therapieoptionen wurden in 3 unabhängigen, randomisierten kontrollierten Studien verglichen [12, 15, 23]. Dabei zeigte sich im Hinblick auf höhere Remissionsraten und eine geringere Krankheitsaktivität nur bei van Vollenhoven et al. (2009) eine Überlegenheit der MTX + bDMARD-Therapie [23]. Moreland et al. (2012) stellten eine geringere radiologische Progression bei der MTX + bDMARD-Therapie fest, während O’Dell et al. (2013) keine signifikanten Unterschiede der Therapieoptionen berichten [12, 15]. Die Jahrestherapiekosten einer bDMARD-Behandlung betragen für das deutsche Gesundheitswesen ca. 11.500–25.000 € pro Patient, während die Kosten für die Dreifachkombination nur wenige Hundert Euro betragen [7]. In Konsequenz zeigen Bansback et al. (2017) in einer Kosteneffektivitätsstudie, dass die MTX + bDMARD-Therapie aufgrund der nur marginal positiven Effekte mit zusätzlichen Kosten von 521.520 $ pro qualitätsadjustiertem Lebensjahr verbunden ist [1].

Die vorliegende Studie weist einige Stärken und Limitationen auf. Routinedaten bieten – besonders vor dem Hintergrund einer stringenten Identifikation der Studienpopulation mittels der Definition von Ausschlusskriterien – die Möglichkeit, Arzneimittelverordnungen im ambulanten Sektor unter Alltagsbedingungen abzubilden. Limitationen ergeben sich vorrangig aus der fehlenden Verfügbarkeit von klinischen Angaben in den Routinedaten, da medizinische Gründe für die geringe MTX-Verordnung nicht nachvollzogen werden können. Andere Studien berichten, dass der Anteil der Patienten, welche die MTX-Therapie dauerhaft aufgrund von Unverträglichkeit oder Toxizität abbrechen müssen ca. 10–15 % beträgt [24, 26]. In dieser Studie führen die Verordnung eines bDMARDs als primäres Einschlusskriterium und die retrospektive Betrachtung der maximal verordneten MTX-Dosierung zu der Annahme eines größeren Anteils von MTX-unverträglichen Patienten. Die Tab. 1 zeigt jedoch, dass die mediane Behandlungsdauer mit 716 Tagen den Beobachtungszeitraum weit übersteigt. Sofern Unverträglichkeiten bei einem Großteil der Patienten vorgelegen hätten, wäre der Einsatz eines bDMARD im Hinblick auf eine leitliniengerechte Behandlung vermutlich früher erfolgt.

Die Aufteilung der Einnahme von oral verordnetem MTX auf 2 oder 3 Tage pro Woche hat möglicherweise positive Effekte auf die Bioverfügbarkeit und Krankheitsaktivität [3, 16]. Auch im vorliegenden Datensatz ist daher davon auszugehen, dass die verordneten MTX-Dosierungen ggf. häufiger als 1‑mal pro Woche eingenommen wurden. Daraus würde sich bei einigen Patienten eine höhere maximale Wochendosierung ergeben.

Zu einem geringen Anteil von 5,4 % weist die Studienpopulation Patienten mit Niereninsuffizienz auf. Im Zusammenhang mit dieser Indikation ist von einer Häufung MTX-induzierter Nebenwirkungen wie Leukopenie, Nephro- und Hepatotoxizität auszugehen [10]. Eine im Vergleich zur gesamten Studienpopulation geringere Maximaldosis entspricht aus der Literatur abgeleiteten Empfehlungen und trägt marginal zu einer geringen Dosierung bei [5].

Nach der im Studienzeitraum gültigen S1-Leitlinie könnte bei bis zu 65,4 % der Patienten vor der Verschreibung eines bDMARDs eine zu geringe MTX-Dosierung verordnet worden sein. Auch unter der Annahme eines höheren Anteils von MTX-unverträglichen Patienten zeigen sich deutliche Hinweise, dass MTX-Dosierungen im ambulanten Versorgungskontext nicht immer hinreichend ausgereizt werden. Die Studie kann dazu beitragen, für eine noch intensivere Ausreizung des Therapiespektrums zu sensibilisieren und ggf. in höherem Maße eine parenterale Applikation in Erwägung zu ziehen.

## Fazit für die Praxis

- Mit ca. 65,4 % ist der Anteil der Patienten, die eine MTX-Dosierung von weniger als 20 mg/Woche verordnet bekamen hoch und ein Indiz für einen nicht immer leitliniengerechten Einsatz der MTX-Dosierung im ambulanten Sektor.
- Es wurde 29,6 % der Patienten MTX im Beobachtungszeitraum ausschließlich oral appliziert. Der Switching-Anteil von oraler zu parenteraler Applikation, bezogen auf die nicht ausschließlich parenteral behandelten Patienten, ist mit 12,4 % ebenfalls gering. Eine Therapie mit parenteral verordnetem MTX sollte in Erwägung gezogen werden, da es dazu beitragen kann, die T2T-Ziele zu erreichen.

## Supplementary Information





## Footnotes

1 Alle *p-*Werte sind in Tabelle S2 des Online-Zusatzmaterials dargestellt.
