# Supplementary material for: Methotrexate treatment before use of biologics in rheumatoid arthritis: Analysis of guideline compliance
Source: Z Rheumatol. 2021 Sep 20;82(7):573–9. [Article in German] doi: 10.1007/s00393-021-01086-0 (PMC10495498; doi:10.1007/s00393-021-01086-0)
Supplement: Supplementary file 1 [file 393_2021_1086_MOESM1_ESM.pdf]

## Zusatzmaterial

**Tabelle S1** Verordnete bDMARDs

| bDMARD | ATC-Code |
|---|---|
| Abatacept | L04AA24 |
| Adalimumab | L04AB04 |
| Anakinra | L04AC03 |
| Certolizumab | L04AB05 |
| Etanercept | L04AB01 |
| Golimumab | L04AB06 |
| Infliximab | L04AB02 |
| Rituximab | L01XC02 |
| Tocilizumab | L04AC07 |

**ATC** Anatomisch-Therapeutisch-Chemisch, **bDMARD** biologic disease-modifying antirheumatic drug

**Tabelle S2** Durchschnittliche MTX-Maximaldosierungen

| | **MTX-Maximaldosierung (mg) MW (SD)** | ***p*-Wert[a,c]** |
|---|---|---|
| **Geschlecht** | | **<0,0001** |
| männlich | 17,1 (±4,8) | |
| weiblich | 14,9 (±5,0) | |
| **Altersgruppe[b]** | 15,7 (±5,3) | 0,1253 |
| **Diagnosegruppe** | | 0,4793 |
| M05 | 16,1 (±5,2) | |
| M06 | 15,9 (±5,1) | |
| M05/M06 | 15,4 (±5,0) | |
| **Krankheitsstatus** | | 0,0705 |
| inzident | 16,5 (±5,3) | |
| prävalent | 15,4 (±5,0) | |
| **Komorbidität: Niereninsuffizienz** | 14,4 (±5,5) | 0,2340 |
| **Subgruppe Leflunomid/Sulfasalazin** | 15,6 (±5,1) | 0,6699 |

[a]Fett gedruckte Werte zeigen statistische Signifikanz zum 5 %-Niveau, [b] Altersgruppe wurde in folgende Klassen eingeteilt: 18-29 Jahre, 30-39 Jahre, 40-49 Jahre, 50-59 Jahre, 60-69 Jahre, 70-83 Jahre, [c] Für die Variablen Altersgruppe und Diagnosegruppe wurde eine einfaktorielle Varianzanalyse genutzt. *p*-Werte der übrigen Variablen basieren auf dem t-Test, **MTX** Methotrexat, **MW** Mittelwert, **SD** „standard deviation", Standardabweichung.

**Tabelle S3** Maximal verordnete MTX-Dosierung im Beobachtungszeitraum der Sulfasalazin/Leflunomid Subgruppe nach Krankheitsstatus

| | **Inzident (n=49)** | | **Prävalent (n=156)** | | **Gesamt (n=205)** | |
|---|---|---|---|---|---|---|
| **MTX-Dosierung** | **n** | **%** | **n** | **%** | **n** | **%** |
| **2,5mg - 7,5mg** | 4 | 8,2 | 11 | 7,1 | 15 | 7,3 |
| **10mg - 12,5mg** | 11 | 22,4 | 42 | 26,9 | 53 | 25,9 |
| **15mg - 17,5mg[a]** | 14 | 28,6 | 46 | 29,5 | 60 | 29,3 |
| **20mg - 22,5mg** | 15 | 30,6 | 47 | 30,1 | 62 | 30,2 |
| **≥25mg[b]** | 5 | 10,2 | 10 | 6,4 | 15 | 7,3 |

[a] Oral verordnete Dosierungen in dieser Gruppe betragen ausschließlich 15 mg, [b] Alle Dosierungen in dieser Gruppe betragen 25 mg, **MTX** Methotrexat.
